# Microglia polarization in nociplastic pain: mechanisms and perspectives

**DOI:** 10.1007/s10787-023-01216-x

**Published:** 2023-04-17

**Authors:** Ahd A. Atta, Weam W. Ibrahim, Ahmed F. Mohamed, Noha F. Abdelkader

**Affiliations:** Department of Pharmacology and Toxicology, Faculty of Pharmacy, Cairo University, Kasr El-Aini St., Cairo, 11562 Egypt

**Keywords:** Nociplastic pain, Fibromyalgia, Microglia activation, M1/M2 microglia polarization, Neuroinflammation, Microgliosis

## Abstract

Nociplastic pain is the third classification of pain as described by the International Association for the Study of Pain (IASP), in addition to the neuropathic and nociceptive pain classes. The main pathophysiological mechanism for developing nociplastic pain is central sensitization (CS) in which pain amplification and hypersensitivity occur. Fibromyalgia is the prototypical nociplastic pain disorder, characterized by allodynia and hyperalgesia. Much scientific data suggest that classical activation of microglia in the spinal cord mediates neuroinflammation which plays an essential role in developing CS. In this review article, we discuss the impact of microglia activation and M1/M2 polarization on developing neuroinflammation and nociplastic pain, besides the molecular mechanisms engaged in this process. In addition, we mention the impact of microglial modulators on M1/M2 microglial polarization that offers a novel therapeutic alternative for the management of nociplastic pain disorders.

**Graphical abstract:**

Illustrating the mechanisms underlying microglia activation in central sensitization and nociplastic pain. LPS lipopolysaccharide, TNF-α tumor necrosis factor-α, INF-γ Interferon gamma, ATP adenosine triphosphate, 49 P2Y12/13R purinergic P2Y 12/13 receptor, P2X4/7R purinergic P2X 4/7 receptor, SP Substance P, NK-1R Neurokinin 1 receptor, CCL2 CC motif ligand 2, CCR2 CC motif ligand 2 receptor, CSF-1 colony-stimulating factor 1, CSF-1R colony-stimulating factor 1 receptor, CX3CL1 CX3C motif ligand 1, CX3XR1 CX3C motif ligand 1 receptor, TLR toll-like receptor, MAPK mitogen-activated protein kinases, JNK jun N-terminal kinase, ERK extracellular signal-regulated kinase, iNOS Inducible nitric oxide synthase, IL-1β interleukin-1β, IL-6 interleukin-6, BDNF brain-derived neurotrophic factor, GABA γ-Aminobutyric acid, GABAR γ-Aminobutyric acid receptor, NMDAR N-methyl-D-aspartate receptor, AMPAR α-amino-3-hydroxy-5-methyl-4-isoxazolepropi-onic acid receptor, IL-4 interleukin-4, IL-13 interleukin-13, IL-10 interleukin-10, Arg-1 Arginase 1, FGF fibroblast growth factor, GDNF glial cell-derived neurotrophic factor, IGF-1 insulin-like growth factor-1, NGF nerve growth factor, CD Cluster of differentiation.

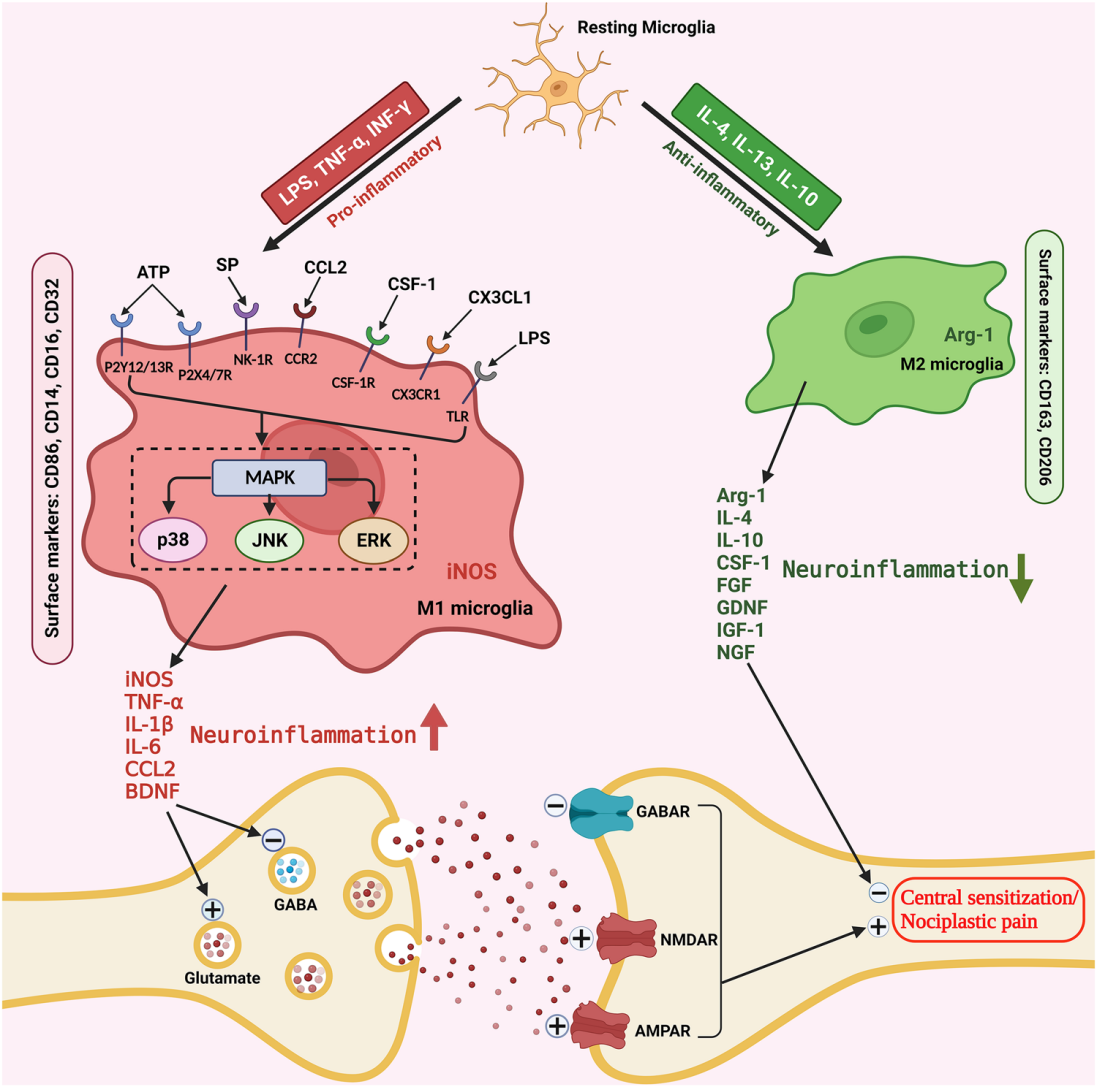

## What is pain?

Pain is a distressing sensation usually caused by noxious or intense stimuli. The International Association for the Study of Pain (IASP) defined pain as “an unpleasant sensory and emotional experience associated with, or resembling that associated with, actual or potential tissue damage” (Raja et al. 2020). Pain is biologically a protecting reflex and a warning symptom for a condition, but it can become a pathologic condition itself and lose its adaptive function, which negatively affects the quality of life (Raffaeli and Arnaudo 2017). Furthermore, pain is the primary motive for doctor consultation in most nations (Debono et al. 2013); being the main symptom in many medical disorders and the main leading cause of patients’ disability and overall functioning impairment (Breivik et al. 2008).

## Classification of pain

Pain can be categorized in many different means according to: (1) the pathogenesis into nociceptive, neuropathic, and nociplastic; (2) the duration into acute and chronic; (3) the etiology into non-malignant and malignant; and (4) the pain anatomic location (Abd-Elsayed and Deer 2019).

### Nociceptive pain

Nociceptive pain is a type of pain brought on by harmful stimuli like tissue injury and inflammation, which stimulate pain receptors known as nociceptors (Yam et al. 2018). Nociceptors are specialized receptors located on the sensory nerve endings of primary afferent nociceptive nerve fibers that are activated by noxious stimuli. Primary afferent nociceptive fibers are classified into three types. (1) Aβ (A-beta) fibers are thickly myelinated, large in diameter, and fast conducting. They have a little activation threshold; thus, they respond to mild pressure, touch, vibration, and hair movement. (2) Aδ (A-delta) fibers are thinly myelinated, small in diameter, and slower in conduction than Aβ fibers. They respond to mechanical and thermal stimuli. They transmit sharp, localized, and rapid pain. (3) C fibers are the smallest primary afferent nociceptive fibers, unmyelinated, and have the slowest conduction. They have a high activation threshold and react to thermal, chemical, and mechanical stimulation. They transmit slow, burning, diffuse, and dull pain (Doody and Bailey 2019). Examples for nociceptive pain are acute trauma, peptic ulcer, and arthritis (Yam et al. 2018).

When nociceptors are stimulated by noxious stimuli, they transform the stimuli into electrical signals, which travel to the spinal cord that in turn delivers the signals up to the higher brain centers. There are four key phases involved in nociception. (1) Transduction: this process occurs in the periphery at site of cell damage that is caused by noxious stimuli. Cell damage releases excitatory neurotransmitters e.g. substance P (SP), prostaglandins (PG), bradykinin (BK), and histamine (H) which stimulate nociceptors. (2) Transmission: the pain impulse travels along afferent nociceptive fibers from the peripheral site, where cell damage occurs, to the spinal cord’s dorsal horn, then up to the brainstem, after that it reaches the thalamus and finally the cerebral cortex. (3) Perception: this phase occurs when the person becomes conscious or aware of the pain. (4) Modulation: during this final phase, the brain alters or modulates the pain by releasing inhibitory neurotransmitters e.g. endorphins, norepinephrine (NE) and serotonin (5-HT) that run down to the spinal cord inhibiting the painful impulses transmission (Ossipov 2012).

### Neuropathic pain

Neuropathic pain is a category of pain developed because of nerve damage or nerve injury rather than nociceptors stimulation. The IASP terms neuropathic pain as “pain initiated or caused by a primary lesion or dysfunction of the nervous system” (Hagen and Rekand 2015). It is a pain condition that is generally chronic and occurs because of progressive nerve disease. Neuropathic pain is often described by patients as a burning, squeezing, or shooting painful sensation. It can happen due to damage anywhere along the nervous system either centrally e.g. pain associated with spinal cord injury and central post-stroke pain, or peripherally e.g. post-herpetic neuralgia and carpal tunnel syndrome (Colloca et al. 2017; Finnerup et al. 2021). The most clinically prevalent peripheral neuropathic pain is that related with diabetes mellitus in which consistent hyperglycemia injures the peripheral nerves throughout the body especially those of the feet and legs (Schreiber et al. 2015; Abdelkader et al. 2022).

### Nociplastic pain

Nociplastic pain develops due to changes in nociceptive processing, probably due to central sensitization (CS), which causes amplification of neural signaling and alteration in pain modulation, ultimately elicits pain hypersensitivity. Nociplastic pain disorders are often coupled with other comorbidities, such as sleep disturbances, fatigue, memory dysfunction, and mood problems. Examples for nociplastic pain are fibromyalgia and irritable bowel syndrome (Fitzcharles et al. 2021). Central sensitization can explain why many people suffer from chronic non-specific pain in the total lack of nerve or tissue damage and a clear activator of nociceptors. The IASP, who was among the first to recognize the CS phenomenon, presented the term “nociplastic pain” in 2017 as the third type of pain, which is distinct from nociceptive and neuropathic pain. Nociplastic pain is described by the IASP as “pain that arises from altered nociception despite no clear evidence of actual or threatened tissue damage causing the activation of peripheral nociceptors or evidence for disease or lesion of the somatosensory system causing the pain” (Kosek et al. 2021). The reported signs and symptoms of CS are present in most nociplastic pain disorders. Moreover, the primary underlying cause of nociplastic pain is CS. Hereafter, patients who have been clinically diagnosed with CS are considered to have a nociplastic pain disorder, such as patients with fibromyalgia, migraine, irritable bowel syndrome, chronic fatigue syndrome, chronic back pain, and non-traumatic neck pain (Nijs et al. 2021). So, we can use nociplastic pain and CS interchangeably. Despite many diseases coming along with nociplastic pain, fibromyalgia is the classical nociplastic pain, which is a long-lasting pain syndrome manifested by fatigue, generalized musculoskeletal pain, depression, and sleep problems (Clauw 2014). Allodynia, hyperalgesia, and spontaneous pain indicate CS.

There are numerous different mechanisms contributing to the pathophysiology of CS: (1) enhanced SP and glutamate levels, (2) potentiation of excitatory signaling of N-methyl D-aspartate (NMDA) receptors and α-amino-3-hydroxy-5-methyl-4-isoxazolepropi-onic acid (AMPA) receptors, (3) over-activated glial cell-derived signals, (4) dysfunction of descending inhibitory pain pathways, and (5) decrease in the inhibitory neurotransmitter gamma aminobutyric acid (GABA) (Harte et al. 2018; Rekatsina et al. 2020). Several studies on fibromyalgia confirmed that higher glutamate levels are essential for the chronic nociplastic pain associated with fibromyalgia (Harris et al. 2009; Valdés et al. 2010; Feraco et al. 2011). The relation between lower levels of GABA and nociplastic pain development and intensity is confirmed in many preceding reports (Foerster et al. 2012; Reckziegel et al. 2016; Cruz-Almeida et al. 2021). Also, increasing levels of SP in nociplastic chronic pain was revealed (Vaerøy et al. 1988; Russell et al. 1994).

## Microglia activation and polarization

Microglia are the brain’s innate immunity cells, which account for about 10% of the cellular population. They are the main phagocytes of the brain. They act as guards of the central nervous system (CNS) and are known for being the first non-neuronal cells to respond against different CNS disorders. Two primary states of microglial cells may be highlighted: resting and active. These states vary based on the needs of the specific tissue. In normal healthy conditions, microglia are in a resting state (Nimmerjahn et al. 2005). However, the cells are actually very mobile and continually scan their environment with their processes (Banati et al. 2004). The resting state of microglia is the prevailing state in the absence of pathological signals in the surroundings. Resting microglial cells transform morphologically and functionally into amoeboid, active microglia when they detect any potentially harmful signals or chemicals or abnormal signaling arriving from neurons and other glial cells (Jurga et al. 2020). Activated and resting microglia are the two opposing morphological forms that lie on each side of a broad spectrum of in-between different phenotypes, according to the amount of activation and the timing of the inflammatory process. Upon activation, microglia can acquire different phenotypes that express diverse intracellular and cell surface markers, release different factors, and perform different functions. Activated microglia are polarized to either the classically activated microglia (proinflammatory, M1) or the alternatively activated microglia (anti-inflammatory, M2). Cytokines, chemokines, prostaglandins, proteases, ferrous iron, and other immunoregulatory components are primarily produced by activated microglia in the CNS (Lan et al. 2017).

### M1 microglia

The microglia polarization towards M1 phenotype is called the classical activation pathway. M1 microglia are the primary responders to an insult. Bacterial-derived products like lipopolysaccharide (LPS), cytokines released by TH1 cells and astrocytes like interferon-γ and tumor necrosis factor-α (TNF-α), and trauma-induced cellular debris all activate the M1 phenotype. M1 microglia release proinflammatory molecules such as TNF-α, inducible nitric oxide synthase (iNOS), interleukin-1β (IL-1β), and interleukin-6 (IL-6) in addition to redox signaling molecules. They also express surface markers e.g. cluster of differentiation (CD) 86, CD14, CD16, CD32, and CD42. Thus, microglial M1 activation is thought to be aggressive causing cytotoxicity, acute immune response, and severe rapid inflammation due to the production of inflammatory chemokines and cytokines as well as reactive oxygen species (Lively and Schlichter 2018; Jurga et al. 2020).

M1 phenotype activation can be measured by detection of surface markers. Levels of CD86, a membrane co-stimulatory receptor in charge of immune cell proliferation, as well as CD16 and CD32, the membrane receptors of Fc region of IgG responsible for induction of inflammatory signals, are raised in activated M1 phenotype. The secreted proinflammatory cytokines (like TNF-α, IL-6, IL-12, IL-18) that are responsible for continuation of inflammation (Kalkman and Feuerbach 2016), and chemokines (such as CCL20, CCL5, CXCL1, CXCL9, CXCL10) that are responsible for recruitment of immune cells can be measured as M1 microglia markers (Könnecke and Bechmann 2013). In addition, iNOS, an enzyme that uses L-arginine to produce NO, serves as a common marker of M1 activation. Its role in microglia is to act against pathogens and tumors through NO synthesis. It also enhances the generation of inflammatory mediators (IL-6) and transcription factors (interferon regulatory factor 1, nuclear factor-kappa B) that are involved in the inflammatory reaction by microglia (Sierra et al. 2014; Bogdan 2015).

### M2 microglia

The microglia polarization towards M2 phenotype is called the alternative activation pathway. It can be thought that switching the activation phenotype towards M2 will have a silencing impact, resulting in reintroducing environmental homeostasis and inducing recovery as opposed to the M1 classical activation pathway. The existence of IL-4, IL-10 or IL-13 induces M2 activation. The latter leads to the release of anti-inflammatory molecules [like IL-4, IL-10, arginase-1 (Arg-1)], growth factors (like insulin-like growth factor I, fibroblast growth factor), neurotrophic mediators (like glial cell–derived neurotrophic factor, brain-derived neurotrophic factor (BDNF), nerve growth factor), and colony-stimulating factor 1 (CSF-1). M2 microglia also expresses surface markers e.g. CD163 and CD206. The consequences of M2 activation is inflammation inhibition, cell proliferation, wound healing, phagocytosis of debris, and homeostasis restoration (Lan et al. 2017; Wang et al. 2019; Jurga et al. 2020).

M2 microglia activation can be measured by detection of surface protein markers. CD163, a hemoglobin scavenger receptor, is responsible for removing oxidative hemoglobin followed by heme degradation by heme oxygenase-1 releasing CO, ferrous ions, and anti-inflammatory byproducts (Etzerodt and Moestrup 2013). CD206 is a receptor found in cellular and endosomal membranes. It is in charge of the processes of endocytosis through recognition of pathogenic polysaccharide chains and glycoproteins (Park et al. 2016; Ohgidani et al. 2017a). The anti-inflammatory cytokines (such as IL-4, IL-10, transforming growth factor beta (TGFβ)) and chemokines (such as CCL2, CCL17, CCL22, CCL24) that inhibit inflammation, are also used as M2 microglia markers (Biswas and Mantovani 2010). Arg-1 is an important marker for M2 activation. This enzyme transforms arginine amino acid into ornithine and urea, which are then used to make proline and polyamides required for healing of wounds and remodeling of tissues (Munder 2009; Quirié et al. 2013).

### Modulation of M1/M2 polarization in nociceptive pain

Nociceptive pain is a warning sign of tissue damage (such as burns, sprains and bone fracture), abnormal muscle tensions, inflammation, obstructions, and increased intraluminal pressure. Immune cells appear to participate significantly in nociceptive pain development. In response to direct or indirect injury to the primary afferent neurons, immune cells, such as spinal microglia or peripheral macrophages, assemble around the neurons. These immune cells generate a variety of proinflammatory and pro-nociceptive factors that interact with nociceptors to cause peripheral sensitization. First, microglia are activated and polarized into M1 macrophages that induce stimulation of the nociceptive fibers, which might be inhibited by M2 microglia later on (Domoto et al. 2021). For example, macrophages are responsible for the chronic pain that happens in rheumatoid arthritis. Rheumatoid arthritis patients exhibit an augmented M1/M2 ratio which encourages inflammation. Peripheral inflammation in rheumatoid arthritis is essential for the activation of microglia. Activated M1 microglia may directly induce pain via producing TNF, IL-6, IL-1β and other proinflammatory cytokines and chemokines that cause synaptic changes centrally and pain hypersensitivity. Hence, chronic nociceptive pain in rheumatoid arthritis is caused by microglia as a consequence of direct neuroinflammation secondary to arthritis itself (Siouti and Andreakos 2019).

### Modulation of M1/M2 polarization in neuropathic pain

Numerous research has emphasized the significance of the neuroimmune process underlying the neuropathic pain development. The ongoing interactions between the immune system and the nervous system cause neuroinflammation, which in turn causes neuropathic pain (Lim and Kam 2020). Macrophages are the key immune cells involved in the development of neuroinflammation. In neuropathic pain disorders, M1/M2 phenotype balance between pro- and anti-inflammatory mediators becomes disrupted and tilted towards M1 macrophages with continuous production of M1 proinflammatory molecules leading to a persistent phase of non-resolving neuroinflammation and the development of long-lasting neuropathic pain (Komori et al. 2011; Kiguchi et al. 2017b; Landis et al. 2018). In context, targeting macrophage polarization has a significant impact on the inflammatory processes, making it a possible approach for treating neuropathic pain (Kiguchi et al. 2017a). For example, in chronic constriction injury rat model of neuropathy, vein wrapping promoted M2 activation with high levels of M2 anti-inflammatory markers (Arg-1, IL-4, IL-10, CD206) resulting in significant increase in pain withdrawal threshold (Hirosawa et al. 2018). Similarly, the increased number of M2 macrophages triggered by IL-4 injection around injured sciatic nerve of mice resulted in a noticeably decreased level of pain behavior (Kiguchi et al. 2015). Another study stated that red light therapy activated M2 macrophages and reduced pain after spinal cord injury (Hu et al. 2016). Also, it has been reported that neuropathic pain brought on by spinal cord injury was relieved by promoting M2 microglia polarization using cerium oxide nanoparticles (Ban et al. 2022).

### M1/M2 imbalance and microgliosis as a primary hallmark of neuroinflammation and a driver of nociplastic pain

It was documented that patients with nociplatsic pain disorders e.g. fibromyalgia show imbalance in normal M1/M2 pattern. In fibromyalgia, serum levels of M1 macrophage markers along with proinflammatory cytokines and chemokines are enhanced, contributing to systemic inflammation (Tripathi et al. 2021). Instead, levels of M2 microglia markers and anti-inflammatory cytokines and chemokines are decreased, resulting in unopposed chronic central inflammatory state (Üçeyler et al. 2006; Sturgill et al. 2014). Classical microglia activation or microgliosis and subsequent chronic neuroinflammation are the pivotal pathophysiological processes connected to the development of chronic nociplastic pain (Albrecht et al. 2019; Donnelly et al. 2020; Hankerd et al. 2022; Álvarez-Pérez et al. 2022).

At the level of spinal cord, microglia activation is modulated by several neuromolecules including adenosine triphosphate (ATP), chemokine CC motif ligand 2 (CCL2), chemokine CX3C motif ligand 1 (CX3CL1, known as fractalkine), colony-stimulating factor 1 (CSF-1), and SP (Johnson et al. 2016). ATP induces microglia activation through stimulation of the purinergic P2Y receptors (P2Y12, P2Y13) and P2X receptors (P2X4, P2X7) (Tsuda et al. 2010; Trang et al. 2012). A prior work suggested that microglia in cases suffering from fibromyalgia were oversensitive to ATP, which induced TNF-α expression. Remarkably, there was a direct correlation between the intensity of fibromyalgia pain and the ATP-induced overexpression of TNF-α (Ohgidani et al. 2017b). The Administration of P2 receptor antagonists, as suramin and TNP-ATP, inhibited neuroinflammation and subsequent pain in previous studies (Wu et al. 2004; Ikeda et al. 2012). Also, CCL2 contributes to microgliosis through chemokine CC Motif Receptor 2 (CCR2) activation (Montague and Malcangio 2017). The inhibition of CCL2 and/or its receptor CCR2 inhibits microglia activation and improves pain in diverse animal models of pain (Padi et al. 2012; Hu et al. 2017; Dubový et al. 2018). Also, CX3CL1 leads to microglia activation via CX3C chemokine receptor 1 (CX3CR1) stimulation. CX3CL1 or CX3CR1 neutralizing antibodies reduces neuroinflammation and attenuates persistent pain sensation (Zhuang et al. 2007; Gao and Ji 2010).

Of note, CSF-1 is crucial for microglia activation in the spinal dorsal horn through binding to its receptor, colony-stimulating factor 1 receptor (CSF-1R), on the microglia contributing to spinal neuroinflammation and induction of pain hypersensitivity (Guan et al. 2016; Yu et al. 2021). As well, microglia Toll-like receptor (TLR) activation contributes to microglia stimulation and neuroinflammation leading to the development of chronic nociplastic pain (Lacagnina et al. 2018; Liu et al. 2022). In a previous study, the administration of the TLR4 antagonists naloxone and naltrexone inhibits TLR4 signaling and the consequential microglia activation resulting in reducing neuroinflammation and alleviating chronic pain (Wang et al. 2016).

Substance *P* participates in the exacerbation of neuroinflammation and its selective receptor, the neurokinin 1 receptor (NK-1R), is highly expressed by M1 microglia. Substance *P* and NK-1R interaction activates microglia and promotes central inflammation that aggravate pain sensation (Wieseler-Frank et al. 2004; Johnson et al. 2016). High levels of SP were evident in the cerebrospinal fluid of patients with fibromyalgia (Stratz et al. 2004).

Taken together, these previously mentioned agonist-receptor interactions lead to activation of microglia intracellular signaling pathways, especially the mitogen-activated protein kinases (MAPKs): p38, c-Jun-N-terminal kinase (JNK) and extracellular signal-regulated kinase (ERK) (Ji et al. 2009, 2018). Inhibitors of p38 and JNK kinases, SE203580 and SP600125 respectively, inhibited microglia activation and neuroinflammation reflected by attenuation of allodynia and hyperalgesia (Xu et al. 2007; Ikeda et al. 2012). Another p38-MAPK inhibitor, dilmapimod, reduced pain in patients suffering from carpal tunnel syndrome (Anand et al. 2011). The activation of MAPKs is a very critical step in the potentiation and persistence of pain due to the generation of multiple proinflammatory cytokines, chemokines, and growth mediators (such as TNF-α, IL-1β, IL6, BDNF, CCL2) resulting in neuroinflammation and increased glutamate release causing NMDAR and AMPAR excitatory synaptic signaling stimulation, in addition to inhibiting GABAR inhibitory signaling in the spinal cord dorsal horn. Therefore, pain is augmented and persists resulting in allodynia and hyperalgesia, which are the main features of CS and also the nociplastic pain (Kawasaki et al. 2008; Ji et al. 2018; Vergne-Salle and Bertin 2021). The high levels of proinflammatory cytokines are consistently detected in patients suffering from different types of chronic nociplastic pain. A previous randomized controlled trial found that plasma levels of TNF-α and IL-6 were elevated in fibromyalgia patients (Ernberg et al. 2018). Moreover, a systematic review reported that TNF-α plasma levels elevated in patients suffering from chronic lower back pain (Morris et al. 2020). In context, the level of proinflammatory cytokine TNF-α was elevated in women suffering from migraine, while the anti-inflammatory cytokine IL-10 level was declined (Oliveira et al. 2017).

## Preclinical and clinical trials focusing on microglial activation and polarization for pain treatment

Microglial polarization shift from the M1 to M2 phenotype is a potential treatment approach for different types of chronic pain as indicated in Table 1. In the early phases of collagen-induced arthritis model, mechanical allodynia and exaggerated spinal nociceptive withdrawal reflexes occurred even before the swelling of hind paw, along with spinal microgliosis and raised IL-1β levels in CSF, suggesting that microglial-induced neuroinflammation contributes to rheumatoid arthritis pain. In rat model of collagen-induced arthritis, administration of microglial inhibitor A-438079 (P2X7 antagonist) decreased the occurrence of mechanical allodynia, reduced IL-1β levels, inhibited microgliosis, in addition to the inhibition of spinal nociceptive withdrawal reflexes (Nieto et al. 2016). In vitro, crotalphine downregulated CD86 expression and enhanced CD206 expression in LPS-treated BV-2 cells, shifting microglial polarization onto the anti-inflammatory M2 phenotype, confirming the neuromodulatory role contributed to crotalphine analgesic action (Lopes et al. 2022). In the bone cancer pain mouse model, spinal cord microglia displayed augmented M1 activation and reduced M2 polarization, as well as up-regulated IL-1β and suppressed IL-10 expression throughout the development of bone cancer pain. Dehydrocorydaline had marked antinociceptive properties coupled with inhibiting M1 phenotype and increasing M2 phenotype of microglia in the spinal cord (Huo et al. 2018).

**Table 1:** Preclinical and clinical trials focusing on microglial activation and polarization for pain treatment

No	Model/disease	Therapeutic approach	Targets/mechanism of action	M1 state	M2 state	Results	References
1	Collagen-induced arthritis pain rat model (in vivo)	A-438079	P2X7 antagonist	Down-regulation: IL-1β	–	Attenuated mechanical allodynia Reduced microgliosis	(Nieto et al. 2016)
2	LPS-induced injury in microglial BV-2 cells (in vitro)	Crotalphine	Decreased LPS-induced CD86 expression and elevated CD206 expression	Down-regulation: CD86	Up-regulation: CD206	Mitigates central neuroinflammation Analgesic effect	(Lopes et al. 2022)
3	Bone cancer pain mouse model (in vivo)	Dehydrocorydaline	Inhibit M1 phenotype, and increase M2 polarization	Down-regulation: iNOS, CD16/32, IL-1β	Up-regulation: Arg-1, CD206, IL-10	Suppressed inflammatory response Antinociceptive effect	(Huo et al. 2018)
4	Chronic constriction injury rat model of neuropathic pain (in vivo)	Kaempferol	Suppression of microglial activation and shifting the M1 to M2	Down-regulation: IL-1β, IL-6, LPS, TNF-ɑ, PGE2	Up-regulation: IL-10	Analgesic action	(Chang et al. 2022)
5	LPS-induced injury in microglial BV-2 cells (in vitro)	kaempferol	Inhibition of TLR4/NF-κB signaling pathway	Down-regulation: IL-1β,TNF-ɑ, CD32, iNOS	Up-regulation: IL-10, Arg-1, CD206	Pain reduction	(Chang et al. 2022)
6	Chronic constriction injury rat model of neuropathic pain (in vivo)	Dual-specificity phosphatase-1 (DUSP1)	Inhibition of the MAPK signaling	Down-regulation	Up-regulation	Increased pain threshold	(Wang et al. 2021)
7	Chronic constriction injury mouse model of neuropathic pain (in vivo)	IL-4	Shifted microglia from the M1 to M2 phenotype	Down-regulation: IL-1β, TNF, iNOS	Up-regulation: IL-10, Arg-1, Ym1	Reduced neuropathy-induced mechanical hypersensitivity Analgesic actions	(Celik et al. 2020)
8	Bone cancer pain rat model (in vivo)	Minocycline	Inhibition of microglia activation	Down-regulation: CD86, IL-1β, TNF-α	Up-regulation: CD206, IL-10	Attenuated mechanical allodynia	(Dai et al. 2019)
9	Spinal cord injury rat model (in vivo)	Propentofylline	Prevention of glial activation	–	–	Increased the mechanical allodynia threshold	(Gwak et al. 2008)
10	Bone cancer pain rat model (in vivo)	Propentofylline	Inhibition of microglia activation	Down-regulation: IL-1β, IL-6, TNF-α	–	Antiallodynic action	(Yao et al. 2011)
11	Peripheral nerve injury rat model (in vivo)	Propentofylline	Suppression of microglial activation	–	–	Attenuated nerve injury-induced mechanical allodynia	(Tawfik et al. 2007)
12	Monoarthritis pain rat model (in vivo)	Propentofylline	Glial inhibitor	–	–	Antinociceptive effect	(Morales et al. 2012)
13	Cancer-induced bone pain rat model (in vivo)	Naringenin	Down-regulation of NF-κB-mediated p65 expression and activation of AMPK/PGC-1α signaling pathway	Down-regulation: CD86, iNOS	Up-regulation: CD206, Arg-1	Antinociceptive effects	(Ge et al. 2022)
14	Spinal nerve ligation pain rat model (in vivo)	Minocycline	Mitigation of microglial activation	Down-regulation: IL-1β, IL-6	Up-regulation: IL-10	Inhibition of neuropathic pain	(Burke et al. 2014)
15	LPS and IFN-γ-induced injury in BV-2 Microglia Cells (in vitro)	Naltrexone	Toll-like receptor 4 antagonism	Down-regulation: iNOS	Up-regulation: CD206	Reduction of neuroinflammation	(Kučić et al. 2021)
16	Chronic compression injury rat model (in vivo)	Botulinum toxin type A	Inhibition of P2X7R expression	Down-regulation: CD68	Up-regulation: CD206	Elevation of pain threshold Relief of neuropathic pain	(Gui et al. 2020)
17	LPS-stimulated HAPI rat microglial cells (in vitro)	Botulinum toxin type A	Inhibition of P2X7R expression	Down-regulation: iNOS, TNF-α, IL-6	Up-regulation: Arg-1, IL-10	Decreased P2X7 protein level Enhancement of M2 polarization	(Gui et al. 2020)
18	Neuropathic pain or fibromyalgia (clinical trial)	Minocycline	Attenuation of microglial activation	–	–	Reduction in number of tender points	(Miwa 2021)
19	Multiple continuous stress rat model of chronic fatigue syndrome and fibromyalgia (in vivo)	Minocycline	Suppression of microglial activation	–	–	Attenuation of allodynia and hyperalgesia	(Yasui et al. 2014)
20	Fibromyalgia (clinical trial)	Naltrexone	Inhibition of microglia activity	–	–	Reduction in fibromyalgia symptoms Improvement in mechanical and heat pain thresholds	(Younger and Mackey 2009)
21	Fibromyalgia (clinical trial)	Naltrexone	Anti-inflammation through modulation of M1/M2 polarization	Down-regulation: IL-1β, IL-6, IL-12, TNF-α	–	Decrease nociception, allodynia, and hyperalgesia Reduction of fibromyalgia-associated pain	(Parkitny and Younger 2017)
22	Widespread muscle pain rat model of fibromyalgia (in vivo)	IL-5	promoting M2 response to counteract the M1 response	–	UP-regulation: CD206	Reduction of hyperalgesia	(Merriwether et al. 2021)
23	Fibromyalgia (clinical trial)	Aquatic exercise	Anti-inflammation through modulation of pro- and anti-inflammatory cytokine production	Down-regulation: TNF-α, IL-6, IL-1β	Up-regulation: IL-10	Improvement of fibromyalgia patients’ quality of life	(Ortega et al. 2012)
24	Reserpine rat model of fibromyalgia (in vivo)	Brilliant Blue G	P2X7R antagonist and inhibition of microglial activation	Down-regulation: IL-1β, IL-18	–	Attenuation of mechanical and thermal hyperalgesia and allodynia	(D’amico et al. 2021)
25	Reserpine rat model of fibromyalgia (in vivo)	Infliximab	Reduced P2X7R expression and its downstream p38-MAPK, and inhibition of microglial activation	Down-regulation: IL-1β, IL-6, TNF-α	–	Reduction of fibromyalgia-associated pain sensitization	(Cordaro et al. 2022)
26	Reserpine rat model of fibromyalgia (in vivo)	Galantamine	Shifted microglia from M1 to M2 phenotype	Down-regulation: iNOS, CD86	Up-regulation: Arg-1, CD163	Analgesic and anti-neuroinflammatory effects	(Atta et al. 2023)
27	Fibromyalgia (clinical trial)	Dextromethorphan	Inhibition of microglial activation	–	–	Mitigation of fibromyalgia-associated pain	(Mueller et al. 2021)
28	Fibromyalgia (clinical trial)	Milnacipran	Reduction of glial activation	–	–	Analgesic properties	(Natelson et al. 2015)
29	Stress-induced irritable bowel syndrome rat model (in vivo)	Minocycline	Inhibition of p38-MAPK pathway and subsequent microgliosis	–	–	Alleviated visceral pain hypersensitivity	(Yuan et al. 2020)
30	Colorectal distension-induced irritable bowel syndrome rat model (in vivo)	Minocycline	Suppression of the activated microglia-dependent inhibition of GABAergic neuronal activity	–	–	Elevation of visceral pain threshold	(Ji et al. 2022)
31	Fibromyalgia (clinical trial)	Cannabidiol	Reduction of microglia activation and M1 polarization	–	–	Analgesic effects	(NCT05283161 2022)

In the chronic constriction injury model of neuropathic pain, the levels of proinflammatory molecules were raised along with development of neuroinflammation due to M1 phenotype activation. Kaempferol alleviated neuropathic pain in rats via suppressing microglia activation and changing its polarization from M1 towards M2 phenotype (Chang et al. 2022). Similarly, dual-specificity phosphatase-1 showed anti-inflammatory properties and alleviated pain in the chronic constriction injury rat model of neuropathic pain induced by through blocking MAPK signaling cascade resulting in switching M1–M2 polarization (Wang et al. 2021). Following chronic constriction injury in mice, IL-4 is thought to ameliorate pain through shifting macrophages from M1 proinflammatory phenotype to M2 anti-inflammatory phenotype, this results in blunting the action of inflammatory mediators and inhibition of pain sensation, in addition to continuous reduction of neuropathy-induced mechanical hyperalgesia, beyond the treatment with IL-4 (Celik et al. 2020).

Microglial polarization shift toward the M1 phenotype is thought to cause the development of bone cancer pain, while minocycline can mitigate the pain of bone cancer through re-shifting microglia polarization towards the M2 phenotype and inhibiting M1 polarization as reflected by augmented expression of M2 microglia marker (CD206) and anti-inflammatory cytokine IL-10, in addition to lowered expressions of M1 microglia marker (CD86) and the proinflammatory cytokines TNF-α and IL-1β (Dai et al. 2019). Propentofylline is a glial cell modulator, acting possibly through direct modulation of microglia to inhibit M1 phenotype and decrease the production of proinflammatory mediators (Sweitzer and De Leo 2011). It attenuates glial activation in the spinal cord dorsal horn and exhibits antinociception in rats suffering from spinal cord injury-induced allodynia (Gwak et al. 2008), bone cancer pain (Yao et al. 2011), and nerve injury-induced allodynia (Tawfik et al. 2007), as well as monoarthritic rats (Morales et al. 2012). Interestingly, promoting microglial polarization to the anti-inflammatory M2 phenotype by naringenin, a natural flavonoid, suppressed microglia-mediated neuroinflammation and attenuated pain sensation in rats with bone cancer pain (Ge et al. 2022).

In rats exposed to spinal nerve ligation-induced cold and mechanical allodynia, minocycline administration inhibited CD11b expression, a microglial activation marker, and reduced the M1 proinflammatory cytokine IL-1β but increased the M2 anti-inflammatory cytokine IL-10 resulting in attenuation of central inflammation and the subsequent pain (Burke et al. 2014). In a study performed in BV-2 microglia cells, low dose of naltrexone induced a shift from the classically activated M1 proinflammatory phenotype to the alternatively activated M2 anti-inflammatory phenotype resulted in marked reduction of proinflammatory cytokines and inhibition of neuroinflammation (Kučić et al. 2021). In chronic compression injury rat model of neuropathic pain, botulinum toxin type A induces microglial M1/M2 polarization towards the anti-inflammatory M2 phenotype through inhibition of P2X7 receptor expression, as confirmed by lower levels of iNOS, IL-6, TNF-α along with higher levels of Arg-1, IL-10 (Gui et al. 2020).

## Targeting microglia as a new therapeutic strategy in treating nociplastic pain

Since increased classical microglial activity contributes to neuroinflammation and CS in patients having chronic nociplastic pain, targeting microglia cells might therefore be a pioneering therapeutic alternative. In context, minocycline is a suppressor of microglia activation and selectively inhibits M1 polarization resulting in decreased proinflammatory molecules production (Kobayashi et al. 2013). In a double blind study, fibromyalgia patients who received minocycline experienced a markedly reduced number of tender points (Miwa 2021). Furthermore, minocycline remarkably attenuated hyperalgesia and allodynia in multiple continuous stress rat models of chronic fatigue syndrome and fibromyalgia by suppressing spinal microglial activation and neural inflammation (Yasui et al. 2014).

The opioid receptor antagonist naltrexone inhibits microglia activation and shows anti-neuroinflammatory properties. Naltrexone was found to mitigate neuroinflammation for treatment of several inflammatory pain disorders including regional pain syndrome and fibromyalgia. In the pilot study of Younger et al., administration of naltrexone at a low dose mitigated the symptoms of fibromyalgia and alleviated mechanical and thermal pain thresholds (Younger and Mackey 2009). In another pilot clinical trial, treatment with naltrexone resulted in marked inhibition of proinflammatory cytokines levels including IL-1β, IL-6 and TNF-α and reduction of fibromyalgia-associated nociplastic pain (Parkitny and Younger 2017).

In an experimental model of fibromyalgia, IL-5 elicits an analgesic effect through inducing macrophage polarization toward M2 anti-inflammatory phenotype (CD206+) serving as a potential strategy to alleviate the pain along with other fibromyalgia-associated somatic symptoms (Merriwether et al. 2021). In a group of female fibromyalgia patients, the M1/M2 imbalance was corrected by an eight-month pool-aquatic exercise program resulting in lower levels of TNFα and increased IL-10 levels, producing anti-inflammatory effect and enhancing the quality of patients’ life (Ortega et al. 2012). In the reserpine-induced fibromyalgia rat model, targeting the microglial P2X7R using its antagonist Brilliant Blue G attenuated microglial activation and consequently the production of pain proinflammatory mediators (IL-1β, IL-18), resulting in inhibiting neuroinflammation and fibromyalgia-mediated pain sensitization (D’amico et al. 2021). Similarly, infliximab administration to rats with reserpine-induced fibromyalgia inhibited microglial stimulation via decreasing the expression of P2X7R and its downstream p38-MAPK, resulting in low levels of IL-1β, IL-6 and TNF-α. Infliximab-treated rats also showed reduced thermal hyperalgesia and mechanical allodynia which reflected an improvement in fibromyalgia-associated symptoms (Cordaro et al. 2022). Also, in the reserpine rat model of fibromyalgia, galantamine administration corrected the M1/M2 balance that was disrupted after reserpine administration and shifted that balance towards the anti-inflammatory M2 phenotype. Galantamine inhibited M1 phenotype which was reflected as a reduction in its markers (iNOS, CD86) and it enhanced M2 polarization which was confirmed by the elevation of its markers (Arg-1, CD163). Galantamine-treated rats showed reduced neuroinflammation and inhibited fibromyalgia-related nociplastic pain (Atta et al. 2023).

In context, dextromethorphan, a well-known antitussive drug, reduced fibromyalgia-associated pain by 30% from baseline levels in a previous clinical trial conducted on 14 women satisfying the fibromyalgia criteria set in 2010 by the American College of Rheumatology. The analgesic effects of dextromethorphan was thought to be mediated via its microglia-modulating properties. Dextromethorphan has been proved to diminish microglia activation and its production of the proinflammatory cytokines iNOS, TNF-α, and IL-6, resulting in inhibiting neuroinflammatory processes (Mueller et al. 2021). Milnacipran, the FDA-approved drug for fibromyalgia treatment, showed analgesic properties in a controlled clinical trial. It inhibited fibromyalgia-associated central inflammatory state through turning off glial activation and subsequent neuroinflammation (Natelson et al. 2015).

Moreover, in the stress-induced irritable bowel syndrome rat model, minocycline infusion reversed stress-induced microgliosis via inhibiting the p38-MAPK pathway, abolishing visceral hypersensitivity as in stress-naive rats (Yuan et al. 2020). In the colorectal distension rat model of irritable bowel syndrome, minocycline curbed the activated microglia-dependent suppression of GABAergic neuronal activity which increased the visceral pain threshold (Ji et al. 2022). In context, cannabidiol has been demonstrated to attenuate pain sensation through its microglia modulatory effects. It reduces M1 neuroinflammatory mediators IL-1β, IL-6, iNOS and CCL2; protecting against microglia-mediated neuroinflammation (Yousaf et al. 2022). Theoretically, cannabidiol can be used to alleviate fibromyalgia-associated pain hypersensitivity. This hypothesis is now under testing in an active clinical trial (NCT05283161 2022).

## Conclusion

Nociplastic pain is established by the IASP as the third pain type in addition to neuropathic and nociceptive pain. The main pathophysiological mechanism of developing nociplastic pain is CS. Fibromyalgia is the ideal disorder to describe nociplastic pain phenomena. Microglia activation and polarization into the M1 phenotype and the subsequent release of proinflammatory chemokines and cytokines are essential for developing neuroinflammation which results in pain hypersensitivity and development of chronic nociplastic pain. In context, microglial modulators may have therapeutic potential through suppressing microglia-mediated neuroinflammation via impeding microglial activation and enhancing its polarization toward the anti-inflammatory M2 phenotype. The current review improves the understanding of the nociplastic pain aspects and discusses the modulatory effect of microglial polarization and microglial modulators as potential new strategies for treatment of nociplastic pain disorders.

## Data Availability

Not applicable.
